# Variants of the *PTPN11* Gene in Mexican Patients with Noonan Syndrome

**DOI:** 10.3390/genes15111379

**Published:** 2024-10-25

**Authors:** Paola Montserrat Zepeda-Olmos, Eduardo Esparza-García, Kiabeth Robles-Espinoza, Juan Ramón González-García, Perla Graciela Rodríguez Gutiérrez, María Teresa Magaña-Torres

**Affiliations:** 1 División de Genética, Centro de Investigación Biomédica de Occidente, Instituto Mexicano del Seguro Social, Guadalajara 44360, Jalisco, Mexico; paolazepeda.genetica@gmail.com (P.M.Z.-O.); kiabethre@gmail.com (K.R.-E.); jrgg_gene@hotmail.com (J.R.G.-G.); pergra.pgrg@gmail.com (P.G.R.G.); 2 Doctorado en Genética Humana, Centro Universitario de Ciencias de la Salud, Universidad de Guadalajara, Sierra Mojada 950, Independencia Oriente, Guadalajara 44340, Jalisco, Mexico; 3 Unidad Médica de Alta Especialidad, Hospital de Pediatría del Centro Médico Nacional de Occidente, Instituto Mexicano del Seguro Social, Belisario Domínguez 735, La Perla, Guadalajara 44360, Jalisco, Mexico; eduardoesparzagenetica@gmail.com

**Keywords:** Noonan syndrome, *PTPN11* gene, Mexican patients

## Abstract

**Background/Objectives:** Noonan syndrome (NS) is a genetic multisystem disease characterized by distinctive facial features, short stature, chest deformity, and congenital heart defects. NS is caused by gene variants of the RAS/MAPK pathway, with *PTPN11* accounting for about 50% of cases. This study aimed to identify *PTPN11* pathogenic variants in Mexican patients with NS to enhance our understanding of the disease in this population. **Methods**: This study included 91 probands and 60 relatives, all of which were clinically evaluated by a geneticist. Sanger sequencing was used to screen the entire *PTPN11* gene. **Results**: Twenty-one previously reported pathogenic variants were identified in 47.3% of the probands. The most frequently occurring were p.Asn308Asp (16.3%) and p.Met504Val (16.3%). Variants p.Tyr279Cys and p.Thr468Met were found exclusively in patients with lentiginosis. Eighty-three percent of patients carried a variant in one of the three exons (3, 8, or 13) where the greatest genetic diversity was observed. Common clinical findings identified in probands included short stature (82%), cardiac anomalies (70.7%), short neck (68.4%), and *pectus excavatum* (63.2%), although features represented by only one patient each were also detected. **Conclusions**: This study confirmed the clinical diagnosis of NS in 43 probands and 11 relatives, and further genetic analysis of the remaining 48 probands is required to identify the causal variant. The genetic and clinical variability observed in our cohort was consistent with reports from other populations, underscoring the importance of comprehensive care for all patients. This research provides the most extensive clinical and molecular characterization of NS in Mexican patients, identifying pathogenic variants of *PTPN11*.

## 1. Introduction

Noonan syndrome (NS) is a relatively common genetic multisystem disease, occurring at a rate between 1 in 2500 and 1 in 1000 live births. NS is characterized by distinctive facial features, a short stature, chest deformity, and congenital heart defects [1,2], such as pulmonary valve stenosis, septal defects, and hypertrophic cardiomyopathy. Other features include cryptorchidism, low weight, a developmental delay of varying degrees, and coagulation defects [3,4].

Diagnosing NS can be challenging due to its variable expressivity and similarities with other disorders, such as Costello syndrome, Cardiofaciocutaneous syndrome, and Legius syndrome. These diseases result from gain-of-function variants of genes that encode proteins of the RAS–mitogen-activated protein kinase (RAS-MAPK) pathway, which plays a critical role in regulating cell differentiation, growth, proliferation, and apoptosis [5]. Additionally, variants that reduce PTPN11 activity cause Noonan syndrome with multiple lentigines (NSML), which shares clinical features with NS [6].

Eleven genes are involved in the etiology of NS (*PTPN11*, *SOS1*, *KRAS*, *RAF1*, *NRAS*, *BRAF*, *LZTR1*, *RRAS2*, *SOS2*, *RIT1*, and *MRAS*), and all are related to the RAS/MAPK signaling pathway [7]. Pathogenic variants of *PTPN11* account for about 50% of cases, followed by *SOS1* (~13%) and *LZTR1* (~8%). NS is generally inherited in an autosomal dominant pattern, with 60%-80% of patients exhibiting de novo variants. This syndrome has a variable expression, but shows complete penetrance [8].

The *PTPN11* gene contains 15 exons that encode protein tyrosine phosphatase non-receptor type 11 (593 amino acids). This protein has two homologous domains (N-SH2 and C-SH2), a protein tyrosine phosphatase domain (PTP), and a C-terminal disorganized domain. In the inactive conformation of PTPN11, the N-SH2 and PTPs interact, resulting in autoinhibition. Upon activation, these domains separate, allowing the PTP to hydrolyze phosphates from target molecules [9,10].

In Mexico, clinical–genetic studies of NS are scarce, even though a complete diagnosis improves the management, surveillance, and outcomes of patients with this condition. Additionally, a segregation analysis is useful for detecting relatives with mild phenotypes, who often remain unacknowledged in terms of having the disease. To date, nine Mexican patients with features consistent with NS have been reported [11,12,13,14], and although the *PTPN11* gene was analyzed in six of them, the causal variant was only found in two related patients (p.Gln79Arg) [14]. In this study, we describe the *PTPN11* gene pathogenic variants in a cohort of patients with clinical characteristics of NS, aiming to further understand this disease among our population.

## 2. Materials and Methods

### 2.1. Study Subjects

A total of 151 individuals were included, comprising 91 probands and 60 related family members. The cases were evaluated by a geneticist, who conducted a comprehensive analysis of the medical history of each patient, encompassing prenatal and perinatal antecedents, psychomotor development, echocardiogram, coagulation studies, and physical examination data. The research protocol was structured in accordance with the Declaration of Helsinki guidelines and received approval from the Research, Ethics, and Safety Committees at our institute.

### 2.2. Molecular Analysis

#### 2.2.1. DNA Extraction

After obtaining informed consent or assent from the probands and their relatives, a blood sample (2 to 4 mL) was collected for genomic DNA extraction using the DTAB-CTAB (dodecyltrimethylammonium bromide–cetyltrimethylammonium bromide) method [15].

#### 2.2.2. Polymerase Chain Reaction

The genetic study was performed by polymerase chain reaction (PCR), followed by Sanger sequencing. To screen the 15 exons and the promoter of the *PTPN11* gene, 14 pairs of primers were designed using the Oligo 6.0 software (Table 1). Various PCR conditions and thermal cycler programs were used to amplify the 14 analyzed fragments, which are described in detail in Table S1. All reactions were carried out at a volume of 10 μL and the amplicons were purified with 0.5 μL of ExoSAP-IT cleanup reagent (Applied Biosystems, Waltham, MA, USA) (the program includes incubation at 37 °C for 15 min then at 80 °C for 15 min) (Cat.78201.1ML). 

#### 2.2.3. DNA Sequencing

The purified PCR products were used for the sequencing reaction, which was carried out in a total volume of 10 μL, containing 100 ng of DNA, 0.5 μL of Ready Reaction Big Dye Terminator Kit v.3.1 (Applied Biosystems, Waltham, MA, USA) (Cat.4337455), 1.5 μL of 5X Buffer, and 2.5 pmol of primer. The thermal cycling program used was as follows: initial denaturation of 96 °C for 4 min, followed by 25 cycles at 96 °C for 10 s, at 55 °C for 5 s, and at 60 °C for 2 min. The sequencing reaction products were purified using columns loaded with 50 μg of Sephadex G-50 medium (Cytiva Life Sciences, Uppsala, Sweden). Capillary electrophoresis was performed on a SeqStudio sequencer, and electropherograms were analyzed both manually and using SnackVar software (Version 2.4.3.) [16] with the reference sequence NM_002834.5. Variants were named according to the nomenclature recommended by the Human Genome Variation Society [17].

#### 2.2.4. Statistical Analysis

The frequency at which allele variants occurred was obtained by direct counting. Fisher’s exact test was used for the comparison of qualitative variables. A *p*-value of less than 0.05 was considered statistically significant.

## 3. Results

### 3.1. Clinical Characteristics

Of the 91 probands with a clinical diagnosis of NS (88 children under 18 years of age and 3 adults), 45 were female and 46 were male, with a median age of 8 years (range: 0–46 years). Pathogenic variants of the *PTPN11* gene were identified in 47.3% (43/91) of the cases, with a median age of 7 years. Of these, 55.8% (24/43) of them were male (42 children and 1 adult). The distribution of NS clinical features in patients with and without *PTPN11* variants is shown in Table 2, Tables S2 and S3 (unfortunately, clinical features were not available in all probands). Characteristics such as a low height for one’s age, heart disease, a short neck, and *pectus excavatum* were noted in over 60% of patients. 

Among all probands, five specific cardiopathies were identified in more than one patient: pulmonary valve stenosis (43.2%), atrial or/and ventricular septal defects (31.3%), hypertrophic cardiomyopathy (20.0%), bicuspid aorta valve (5.0%), and arrhythmia (5.0%). Seven other heart diseases were only detected in one patient each: aortic stenosis, mitral stenosis, partial anomalous pulmonary venous connection, interatrial aneurysm, double-chambered right ventricle, the transposition of the great arteries, and tetralogy of Fallot. Additionally, 25.6% of patients presented two or three of these conditions in concomitance. A comparison between variant-positive and variant-negative *PTPN11* patients showed significant differences between two variables. Easy bruising was more frequent in variant-positive patients (23.6% vs. 7.5%; *p* = 0.03), while *pectus excavatum* was more common in variant-negative patients (63.2% vs. 83.3%, *p* = 0.04).

Currently, there is no specific pharmacological treatment for NS. However, patients often use medications to manage their associated medical conditions. In the interrogatory phase, 24.2% of the patients with *PTPN11* pathogenic variant expressed having taken cardiac drugs, 12.1% having taken neuro-psychiatric drugs, and 48.5% having received growth hormone therapy. Furthermore, ten patients underwent cardiac surgery and seven underwent orchidopexy.

### 3.2. Molecular Analysis

We identified heterozygous pathogenic variants in 43 out of 91 probands included in this study. A total of 21 different missense variants were detected, with p.Asn308Asp and p.Met504Val being the two most frequent, together representing 32.6% of cases. Moreover, two other recurrent variants, p.Ala72Ser (7%) and p.Thr468Met (7%), were found in three patients each.

The highest genetic heterogeneity was observed in exon 3, with nine different variants distributed among 14 patients, and the most common one, p.Ala72Ser, was present in 3 of them. Notably, 95.2% of variants affected amino acids situated in N-SH2 and PTPs, whose function is to modulate the activity of PTPN11 (Table 3).

### 3.3. Segregation Analysis

In order to determine if a genetic variant was inherited or de novo and to provide appropriate genetic counseling, family segregation analysis was performed in 60 individuals related to 28 probands (regrettably, this study could not be completed in 15 cases). The results revealed that 18 patients had a de novo variant, while 10 inherited the variant (7 maternally and two paternally, and in 1 proband the parents were not available and so a brother was tested, who also turned out to have the variant). This analysis allowed us to detect 11 cases with NS (9 parents and 2 siblings), bringing the total number of patients with a clinical and molecular diagnosis of NS to 54 (Table S4).

## 4. Discussion

We report the findings of 91 unrelated patients who were analyzed for variants of the *PTPN11* gene. Heterozygous pathogenic variants were detected in 43 of the 91 probands, representing a detection rate of 47.3%. This aligns with previous reports, which suggest that approximately 50.0% of NS patients have a *PTPN11* variant [18]. For the remaining 52.7% of probands, the analysis of other genes is required to identify the causal variant and confirm the clinical diagnosis. In the absence of such findings, differential diagnosis should be considered.

In NS, variable expressivity contributes to the underdiagnosis of cases with mild clinical phenotypes. Through segregation analysis, we identified 11 relatives with NS, 9 of whom were unaware of their condition. These findings highlight the importance of screening both affected and apparently unaffected consanguineous relatives when a pathogenic variant is detected in a proband. This approach increases the rate of detection of NS patients and allows for more accurate follow-up treatment and genetic counseling.

The distribution of variant-positive patients by sex was comparable between males (55.8%) and females (44.2%, *p* = 0.28), as expected, given the inheritance pattern of the syndrome. Investigations into associations among clinical characteristics in variant-positive and variant-negative patients revealed that two variables reached statistical significance. First, easy bruising was more frequent in variant-positive patients (*p* = 0.03); this association has been reported previously [19,20], as PTPN11 is a negative regulator of thrombus stability [21]. Some patients may experience coagulation problems even when their evaluation parameters are normal. Therefore, the risk of bleeding must be considered in patients undergoing surgery [22]. The second variable, *pectus excavatum*, was more common in variant-negative patients (*p* = 0.04), a finding not reported in other studies.

The most frequent clinical manifestations (>60%) in patients with *PTPN11* pathogenic variants were a short stature, heart disease, a short neck, *pectus excavatum*, and cryptorchidism. 

Having a short stature is a clinical feature of NS, with a comparable frequency in Mexican patients to that reported in Chinese (32/39 vs. 37/50; *p* = 0.36) [23] and Northern European patients (32/39 vs. 39/51; *p* = 0.52) [23,24]. However, the rate was significantly different from that in Indian patients (32/39 vs. 43/107, *p* < 0.0001) [18]. Growth hormone treatment has proven to be safe and effective, regardless of the presence of a growth hormone deficiency, although long-term studies are necessary [25]. In this study, 48.5% of patients with the *PTPN11* pathogenic variant had received growth hormone, but treatment response analysis could not be performed due to data insufficiencies. On the other hand, psychosocial factors may impact well-being more than height itself, suggesting that treatment should as well [26].

The prevalence of heart disease in our patients correlates with that found in Chinese patients (29/41 vs. 41/50; *p* = 0.20) [23], but it was lower than that in Turkish patients (29/41 vs. 11/11; *p* = 0.04) [27], which was potentially due to undiagnosed milder cases. The PTPN11 protein plays a crucial role in semilunar valvulogenesis, influencing mesenchymal transformation, cell proliferation, and subsequent valve remodeling, all of which are mediated by epidermal growth factor signaling [24]. Most studies have observed that pulmonary valve stenosis is more common in patients with NS who carry a *PTPN11* variant [28]. We also observed this trend (51.2% vs. 35.0%, *p* = 0.18), although the difference was not significant. This condition often manifests in early childhood (0–6 years), and six of our patients remain at risk as they are under 6 years of age. Other conditions, such as Mowat–Wilson and Alagille syndromes, should be considered in patients without a *PTPTN11* variant [29]. However, the prevalence of this cardiopathy in our study is consistent with that in other populations (Chinese 42.0% and Indian 35.3%) [18,23].

Having a short neck was a common observation in this study, with a similar frequency to that observed in Russian patients (26/38 vs. 66/107; *p* = 0.45) [30]. This characteristic appears to have no medical complications. 

*Pectus excavatum* is a significant indicator of NS that can lead to cardiological, respiratory, and body image disturbances. Therefore, proper evaluation and surgical correction, if necessary, are recommended [31]. In this study, 63.2% of patients with a *PTPN11* variant had *pectus excavatum*, and none of them underwent either surgical intervention or psychological assessment. Interestingly, its frequency was significantly higher in our cohort compared to Chinese patients (24/38 vs. 16/50, *p* = 0.005) [23] but was similar to that found in Indian patients (24/38 vs. 57/107 *p* = 0.29) [18].

Cryptorchidism was observed in 73% (16/22) of our patients, which was similar to the rate in Chinese (17/27, *p* = 0.46) and Italian patients (n = 5/8, *p* = 0.58) [23,32] but higher than that in Russian patients (n = 27/63, *p* = 0.01) [30]. The etiology of cryptorchidism remains unclear, although it is associated with several genetic syndromes [33] and risk factors such as smoking, alcohol consumption, and gestational diabetes [34]. Early corrective surgery reduces the risk of developing cancer [35].

We identified 21 pathogenic variants of the *PTPN11* gene in 43 probands; all of these had previously been reported. Notably, 83.7% (36/43) of the patients’ variants were clustered in three exons: 3, 8, and 13. This distribution has been found in other populations, including Chinese (94%) [23], Greek (81.5%) [36], Indian (79.5%) [18], Northern European (83.3%) [24], and South American (86%) [37]. In fact, when considering our entire cohort, screening just these three exons could establish a molecular diagnosis in 39.6% (36/91) of patients, making this an effective strategy with a favorable cost–benefit ratio.

The genetic heterogeneity of NS was evident, with most variants occurring infrequently. We detected two variants, each with a 16.3%. The first, p.Asn308Asp, has been observed in several populations, with ranges varying from 16.3% to 43%. The second, p.Met504Val, is less common, with percentages from 4.2% to 16.3% [23,24,36,38]. We also found that 52.4% of the variants were found only once per proband, which is comparable to findings in Chinese (40%) and Northern European patients (53.2%) [23,24].

Variants that reduce the catalytic activity of PTPN11 are associated with NSML. We detected 9 patients with such variants: 6 probands (2 with p.Tyr279Cys, 3 with p.Thr468Met, and 1 with p.Gln510Glu) and 3 relatives (2 with p.Tyr279Cys and 1 with p.Thr468Met). Six of these patients (three probands and three relatives) exhibited lentigines, while two probands who were less than two years old had not yet shown this characteristic, which typically appears between the ages of four and five [39]. No information was available regarding one patient (Supplementary Table S2). 

Causative variants of NS (e.g., p.Asp61Gly, p.Ala72Gly, p.Thr73Ile, p.Tyr279Cys, p.Thr468Met, p. Arg498Trp, p.Ser502Ala, and p.Gly506Pro) have been associated with an increased risk of developing cancer, including myeloproliferative disorders and juvenile myelomonocytic leukemia [40]. Consequently, it is recommended that infants with NS undergo a physical examination and complete blood count every 3–6 months during their first five years of life [41]. In our cohort, 13 patients carried variants linked to cancer; fortunately, none developed malignancies.

## 5. Conclusions

This research provides the most extensive clinical and molecular characterization of Noonan syndrome in Mexican patients, identifying 21 pathogenic variants of the *PTPN11* gene. We confirmed the diagnosis in 43 probands and 11 relatives; however, exome studies are needed for the 48 probands who did not have a variant in *PTPN11.* Great clinical variability was observed, which highlights the relevance of long-term follow-ups to monitor complications in each patient and ensure comprehensive care from various specialists, including cardiologists, endocrinologists, psychologists, and dermatologists. With clinical and molecular diagnosis, patients will receive accurate genetic counseling. In addition, family segregation analyses will enhance the detection of affected individuals. 

## Figures and Tables

**Table 1:** Primers used to amplify the *PTPN11* gene exons.

Exon	Primer Sequence (5′-3′)	Fragment Lenght (bp)
Promoter-1F	CTGCACAGTCTCCGGGATC	397
Promoter-1R	GGCAGGAAATGAATGGGGAC	
2F	CAGGGAAGGTCTTGATTTG	328
2R	GCTATCCAAGCATGGTTTTAC	
3F	GGTAAAATCCGACGTGGAAG	394
3R	ACAGTCACAAGCCTTTGGAG	
4F	GTGTTTAGGAGAGCTGACTG	369
4R	AATGGTGTCTGTCTTCTGTC	
5F	ACCCAGCCTATTATCTGTC	456
5R	CTGTACTCCAGACTGGGTG	
6F	GGTCCTATGAACCCTCTGTC	320
6R	CCAAACACAAGAGCAACTTC	
7F	GAAGTAATGCTGATCCAGGC	279
7R	CCGATGTGCTAACAAGAGC	
8–9F	GGGGAGTAACTGATTTGAAC	570
8–9R	CTAGTCCCTTTTTTCCAGAG	
10F	CCATGTTGGTGGTTATTAAG	364
10R	ATGGCTACTGAATCAATGAG	
11F	AGGACCTTTCAGTGGAACC	373
11R	AAGAGCTAGGAGTGGGTAGG	
12F	AAAGCCCTATGCTTTTTGC	331
12R	TCCCATTCAACCTCTCTTC	
13F	AGACTAAATTAGCATTGTCTCTGAG	360
13R	CAAACAGTTGTCTATCAGAGCC	
14F	CCTTGAGAAGGTGAATCCC	525
14R	GATTACAGGCGTTAGCCAC	
15F	GCGTTATTTCACTTCTGCC	448
15R	TACAGGGAGAGGAAAAAGG	

PCR conditions and thermal cycler programs are described in detail in Table S1.

**Table 2:** Clinical characteristics of Noonan syndrome patients with and without *PTPN11* gene variants ^1^.

Clinical Variables	Variant-Positive		Variant-Negative	
	n ^2^	%	n ^2^	%
Low height for age	32/39	82.1	28/41	68.3
Cryptorchidism	16/22	72.7	9/20	45.0
Heart disease	29/41	70.7	26/40	65.0
Short neck	26/38	68.4	29/42	69.0
*Pectus excavatum*	24/38	63.2	35/42	83.3 ^3^
Low weight for age	22/39	56.4	17/41	41.5
Growth hormone treatment	16/33	48.5	16/33	48.5
Pulmonary valve stenosis	21/41	51.2	14/40	35.0
Sparse eyebrows	16/38	42.1	16/40	40.0
Speech delay	16/39	41.0	18/39	46.2
Microcephaly	13/40	32.5	12/41	29.3
Global developmental delay	13/40	32.5	11/39	28.2
Gross motor delay	13/40	32.5	13/39	33.3
Prolonged PTT (>37.3 seg)	10/31	32.3	9/34	26.5
Cardiac pharmacological treatment	8/33	24.2	11/33	33.3
Heart septal defects	11/40	27.5	14/40	35.0
Easy bruising	10/38	26.3	3/40	7.5 ^4^
Respiratory distress at birth	9/40	22.5	12/38	31.6
Preterm birth (<37 gw)	9/40	22.5	12/39	30.8
Cardiomyopathy	9/40	22.5	7/40	17.5
Deep creases	8/38	21.1	6/40	15.0
Cognitive delay	8/40	20.0	10/39	25.6
Intellectual disability (mild to moderate)	8/40	20.0	11/40	27.5
Learning disabilities	8/40	20.0	12/39	30.8
Polyhydramnios in prenatal ultrasound	7/38	18.4	8/36	22.2
*Pterigium Colli*	7/38	18.4	12/42	28.6
Fine motor delay	7/40	17.5	10/39	25.6
Café au lait macules	6/38	15.8	4/37	10.8
Sparse hair	6/38	15.8	4/40	10.0
Prolonged prothrombin time (>13.4 seg)	4/27	14.8	8/32	25.0
Neuro-psychiatric pharmacological treatment	4/33	12.1	4/33	12.1
Refraction problems	5/38	13.2	4/40	10.0
*Pectus carinatum*	5/39	12.8	2/40	5.0

PTT: partial thromboplastin. ^1^ A complete list of all clinical characteristics can be found in Table S2; here, we only present the most frequently occurring ones (>10%). ^2^ Unfortunately, not all medical records were complete. *p*-values of variables with significantly different distribution between both groups: ^3^ *p* = 0.04 and ^4^ *p* = 0.03.

**Table 3:** Pathogenic variants of the PTPN11 gene in Mexican patients with Noonan syndrome.

Exon	Affected Domain	Reference SNP	Nucleic Change	Codon Alteration	Aminoacid Change	No. Patients
2	N-SH2	rs397507501	c.124A>G	ACA>GCA	p.Thr42Ala	1
3	N-SH2	rs397507505	c.172A>G	AAC>GAC	p.Asn58Asp	2
3	N-SH2	rs397507509	c.179G>C	GGT>GCT	p.Gly60Ala ^a^	1
3	N-SH2	rs121918461	c.182A>G	GAT>GGT	p.Asp61Gly	1
3	N-SH2	rs121918460	c.184T>G	TAC>GAC	p.Tyr62Asp ^a^	1
3	N-SH2	rs121918459	c.188A>G	TAT>TGT	p.Tyr63Cys ^a^	2
3	N-SH2	rs397507511	c.205G>C	GAG>CAG	p.Glu69Gln ^b^	1
3	N-SH2	rs121918453	c.214G>T	GCC>TCC	p.Ala72Ser	3
3	N-SH2	rs121918462	c.218C<T	ACT>ATT	p.Thr73Ile	1
3	N-SH2	rs121918466	c.236A>G	CAG>CGG	p.Gln79Arg ^a^	2
4	C-SH2	rs397507520	c.417G>C	GAG>GAC	p.Glu139Asp	1
7	PTP	rs121918456	c.836A>G	TAT>TGT	p.Tyr279Cys ^b^	2
7	PTP	rs397507529	c.844A>G	ATC>GTC	p.Ile282Val	1
8	PTP	rs121918463	c.854T>C	TTT>TCT	p.Phe285Ser	1
8	PTP	rs121918455	c.923A>G	AAT>AGT	p.Asn308Ser	2
8	PTP	rs121918455	c.922A>G	AAT>GAT	p.Asn308Asp ^b^	7
12	PTP	rs121918457	c.1403C>T	ACG>ATG	p.Thr468Met ^a^	3
13	PTP	rs397507543	c.1502G>A	AGG>AAG	p.Arg501Lys	1
13	PTP	rs397507545	c.1507G>A	GGG>AGG	p.Gly503Arg	2
13	PTP	rs397507547	c.1510A>G	ATG>GTG	p.Met504Val	7
13	PTP	rs397507549	c.1528C>G	CAG>GAG	p.Gln510Glu	1
					Total	43

Variants observed through segregation analysis in the studied relatives, where ^a^ indicates a variant detected in one patient and ^b^ indicates a variant detected in two patients.

## Data Availability

The original contributions presented in the study are included in the article and Supplementary Materials, further inquiries can be directed to the corresponding author.

## Supplementary Materials

The following supporting information can be downloaded at: https://www.mdpi.com/article/10.3390/genes15111379/s1. Table S1: PCR conditions and thermal cycler programs used to amplify the 14 analyzed fragments. Table S2: Clinical characteristics of Noonan syndrome patients with and without *PTPN11* gene variants. Table S3: Clinical and genetic characteristics of Mexican Patients with Noonan syndrome and *PTPN11* variants. Table S4: Segregation analysis of the probands’ relatives.
