# Supplementary material for: Variants of the PTPN11 Gene in Mexican Patients with Noonan Syndrome
Source: Genes (Basel). 2024 Oct 25;15(11):1379. doi: 10.3390/genes15111379 (PMC11593480; doi:10.3390/genes15111379)
Supplement: Supplementary file 1 [file genes-15-01379-s001.zip › genes-3245484-supplementary/Supplementary tables/Table S1..pdf]

Table S1. PCR conditions and thermal cycler programs used to amplify the 14 analyzed fragments.

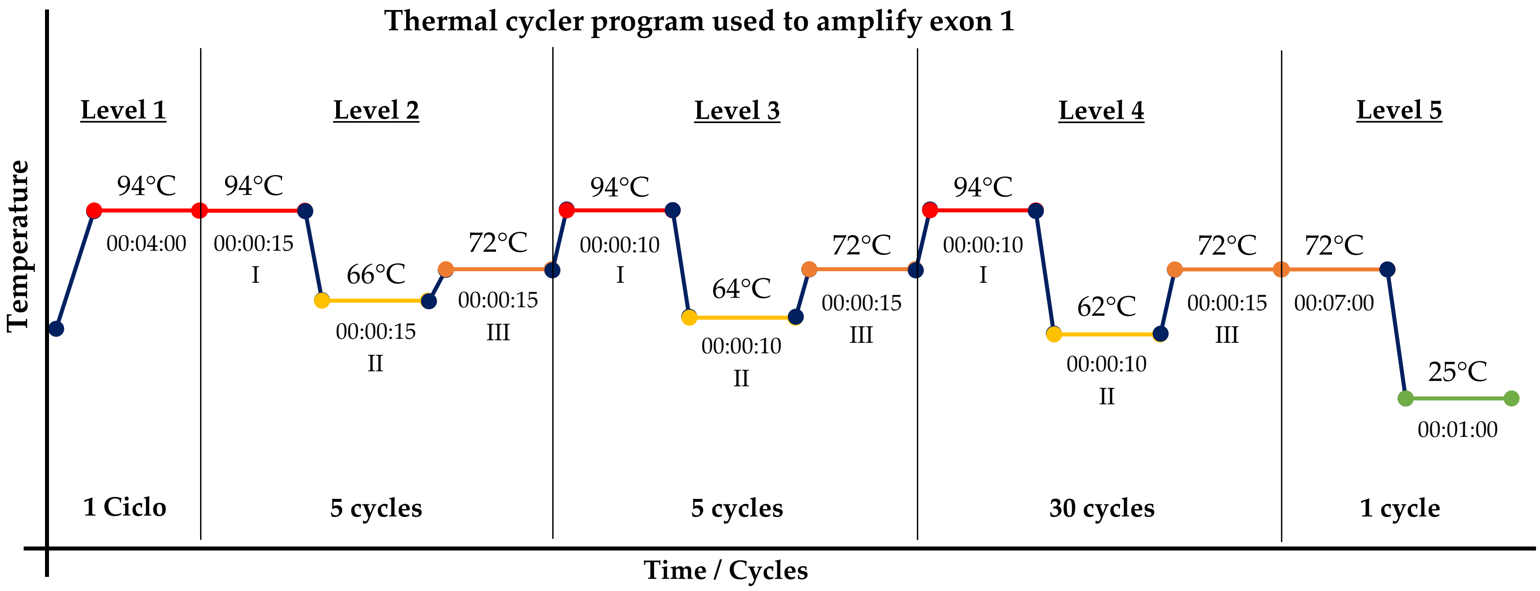

I. Denaturation **II.** Hybridization **III.** Extension

All PCR reactions were carried out in a total volume of 10 uL. The conditions for the exon 1 were as follows: 50-100 ng of DNA, 2.5 pmol of each primer, 3.0mM $MgCl_2$, 5% DMSO, 0.2mM of each dNTP, 1X buffer (10X PCRX; Invitrogen P/N52395), 0.25 units of Platinum Taq polymerase (Invitrogen REF 10966-030).

Thermal cycler program used to amplify exons 2, 3, 4, 5, 6, 7, 8-9, 10, 11, 12, 13, and 15

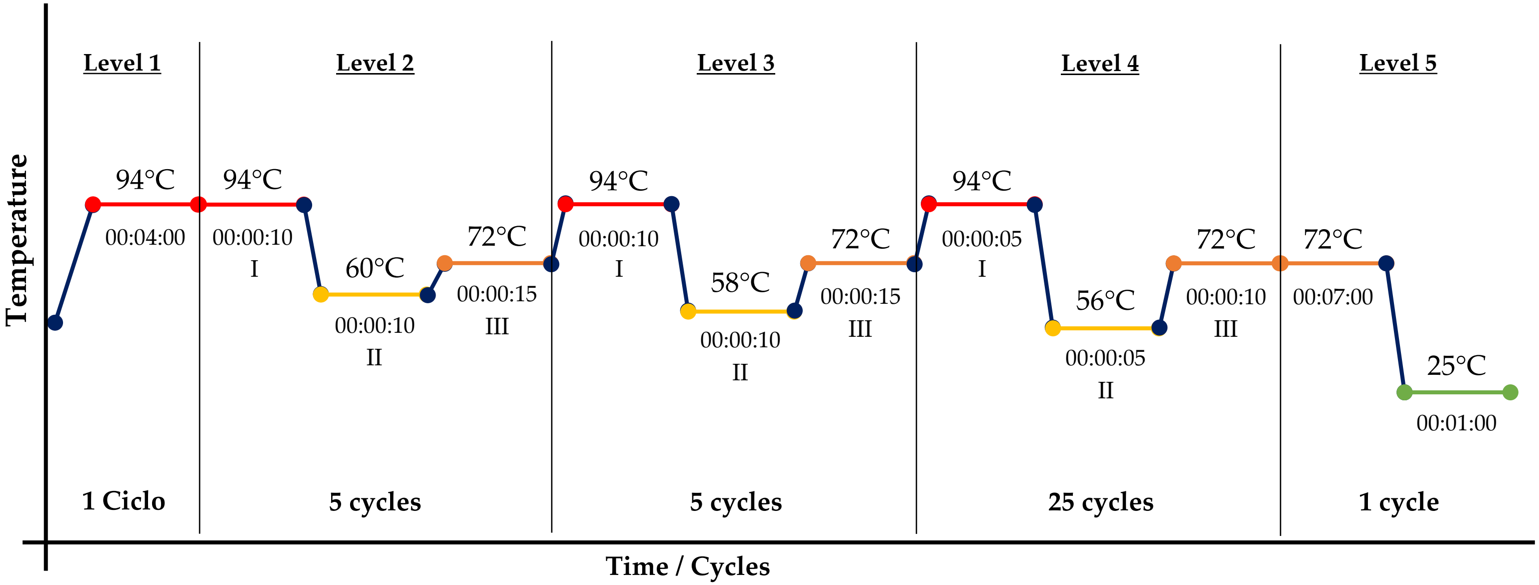

I. Denaturation **II.** Hybridization **III.** Extension

All PCR reactions were carried out in a total volume of 10 uL. The conditions for exons 4, 6, 7, 8-9, 10, 11, 12, 13, and 15 were as follows: 50-100 ng of DNA, 2.5 pmol of each primer, 3.0mM of $MgCl_{2,}$ 0.2mM of each dNTP, 1X buffer (10X PCR; Invitrogen P/N Y02028b), 5% DMSO, 0.05 units of Taq polymerase (thermos scientific REF EP0404). The conditions for exons 2 and 3 were the same, except for the use of 2mM MgCl2 and the exclusion of DMSO. For exon 5, Taq Platinum (0.25 U, Invitrogen REF 10966-030) was used, while the other reagents remained the same.

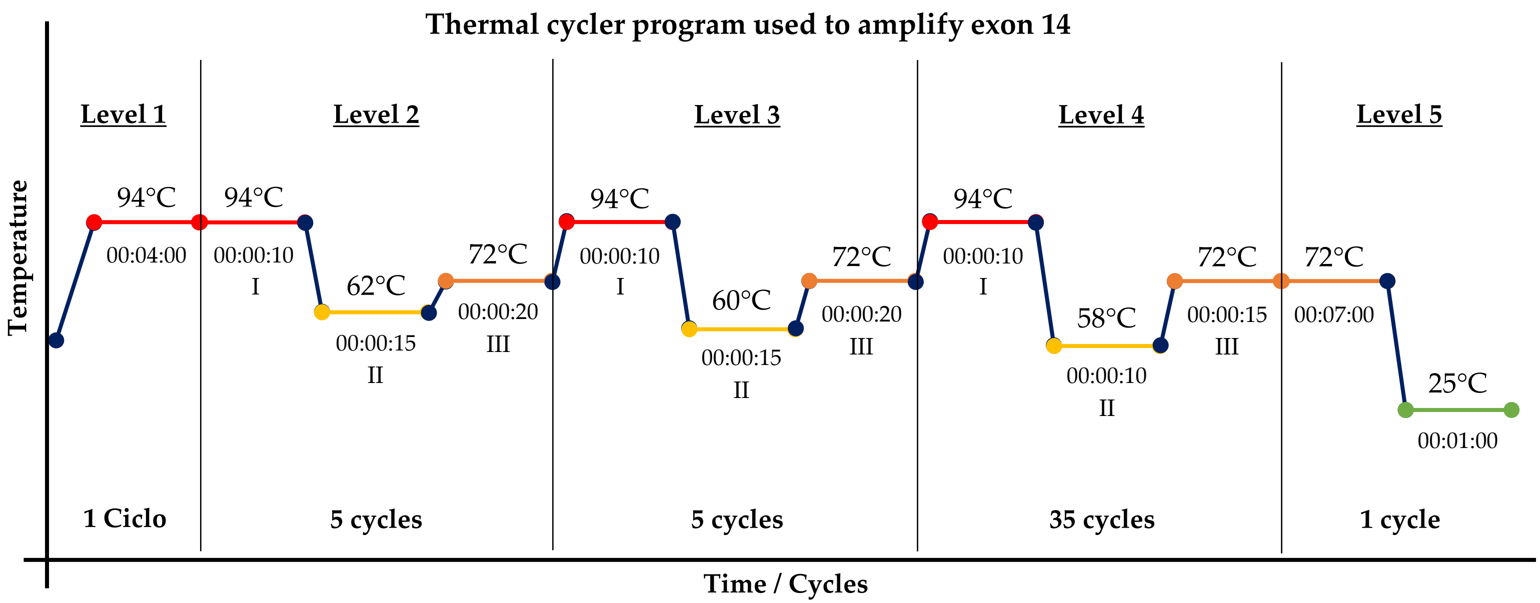

I. Denaturation **II.** Hybridization **III.** Extension

All PCR reactions were carried out in a total volume of 10 uL. The conditions for the exon 14 were as follows: 50-100 ng of DNA, 2.5 pmol of each primer, 3.0mM of $MgCl_2$, 5% DMSO, 0.2mM of each dNTP, 1X buffer (10X PCRX; Invitrogen P/N52395), 0.25 units of Platinum Taq polymerase (Invitrogen REF 10966-030)
