# Supplementary material for: Variants of the PTPN11 Gene in Mexican Patients with Noonan Syndrome
Source: Genes (Basel). 2024 Oct 25;15(11):1379. doi: 10.3390/genes15111379 (PMC11593480; doi:10.3390/genes15111379)
Supplement: Supplementary file 1 [file genes-15-01379-s001.zip › genes-3245484-supplementary/Supplementary tables/Table S2. Clinical characteristics of Noonan Syndrome patients with and without PTPN11 gene variants.pdf]

Table S2. Clinical characteristics of Noonan Syndrome patients with and without *PTPN11* gene variants

**Clinical variable**	**Patients with pathogenic variant in *PTPN11***		**Patients without pathogenic variant in *PTN11***		Value P^3
	**n[1]**	**n[2]**	**n[1]**	**n[2]**	
Increased nuchal translucency in prenatal ultrasound	2	38	0	34	0.49
Cystic hygroma in prenatal ultrasound	1	38	1	35	1
Hydrops fetalis in prenatal ultrasound	1	38	0	35	1
Polyhydramnios in prenatal ultrasound	7	38	8	36	0.77
Feeding difficulties	4	40	5	38	0.73
Hypotonia	3	40	1	38	0.61
Respiratory distress at birth	9	40	12	38	0.44
Laryngomalacia	1	40	0	38	1
Preterm birth (<37 gw)	9	40	12	39	0.45
Lymphatic dysplasia in prenatal ultrasound	0	40	2	38	0.23
Renal abnormalities	0	40	1	38	0.48
Low weight for age	22	39	17	41	0.26
Low height for age	32	39	28	41	0.2
Microcephaly	13	40	12	41	0.81
Macrocephaly	1	40	2	39	0.61
Cognitive delay	8	40	10	39	0.6
Speech delay	16	39	18	39	0.82
Global developmental delay	13	40	11	39	0.8
Intellectual disability (mild to moderate)	8	40	11	40	0.6
Learning disabilities	8	40	12	39	0.31
Fine motor delay	7	40	10	39	0.42
Gross motor delay	13	40	13	39	1
Muscular weakness	1	40	1	39	1
Seizures	1	40	4	40	0.35
Central nervous system anomalies	2	39	1	39	1
Attention Deficit Hyperactivity Disorder	4	40	6	39	0.51
Anxiety	2	40	2	39	1
Heart disease	29	41	26	40	0.63
Pulmonary valve stenosis	21	41	14	40	0.18
Septal defect	11	40	14	40	0.63
Bivalve aorta	1	40	3	40	0.61
Cardiomyopathy	9	40	7	40	0.78
Arrhythmia	1	40	3	40	0.61
Growth hormone treatment	16	29	16	32	0.79
Psychiatric pharmacological treatment	4	29	9	32	0.21
Cardiac pharmacological treatment	8	29	11	32	0.59
Levothyroxine treatment	1	29	2	32	1
Café au lait macules	6	38	4	37	0.73
Sparse hair	6	38	4	40	0.51
Sparse eyebrows	16	38	16	40	1
Sparse eyelashes	1	38	1	40	1
Alopecia	2	38	3	40	1
Keratosis pilaris	1	37	2	40	1
Melanocytic nevus	0	38	2	40	0.49
Lentiginosis	3	38	0	40	0.11
Skin hyper elasticity	0	38	1	40	1
Deep creases	8	38	6	40	0.56
Hemangioma	2	38	4	40	0.67
Hyperhidrosis	0	38	1	40	1
Nail dysplasia	0	38	3	40	0.24

Easy bruising	10	38	3	40	0.034
Bleeding tendency	3	38	3	40	1
Thrombocytopenia	0	35	2	41	0.49
Prolonged prothrombin time (>13.4 seg)	4	27	8	32	0.51
Prolonged partial thromboplastin time (>37.3 seg)	10	31	9	34	0.78
Joint Hypermobility	2	38	4	40	0.67
Short neck	26	38	29	42	1
Pterygium Colli	7	38	12	42	0.3
Pectus excavatum	24	38	35	42	0.047
Pectus carinatum	5	39	2	40	0.26
Scoliosis	2	38	0	40	0.23
Hip dysplasia	1	38	0	40	0.48
Hearing impairment	0	38	1	40	1
Ptosis	4	38	10	41	0.14
Strabismus	1	38	3	40	0.61
Refraction problems	5	38	4	40	0.73
Cryptorchidism	16	22	9	20	0.11

n^1 Number of patients who presented the clinical characteristic

n^2 Number of patients who underwent analysis of the clinical characteristic

[3] P-value obtained from the comparison between the two groups of patients: variant + and variant -
