# Supplementary material for: Variants of the PTPN11 Gene in Mexican Patients with Noonan Syndrome
Source: Genes (Basel). 2024 Oct 25;15(11):1379. doi: 10.3390/genes15111379 (PMC11593480; doi:10.3390/genes15111379)
Supplement: Supplementary file 1 [file genes-15-01379-s001.zip › genes-3245484-supplementary/Supplementary tables/Table S4.Segregation analysis of the probands' relatives.pdf]

Table S4. Segregation analysis of the probands' relatives

No. Family	Variant of the proband	Mother	Father	Sibling 1	Sibling 2	Inheritance
1	p.Thr468Met	normal	normal			*de novo*
2	p.Gln79Arg	normal	normal			*de novo*
3	p.Asn58Asp	normal	normal			*de novo*
4	p.Asn308Asp	normal	normal			*de novo*
5	p.Asn308Ser	normal	normal			*de novo*
6	p.Met504Val	normal	normal			*de novo*
7	p.Gly503Arg	normal	normal			*de novo*
8	p.Thr73Ile	normal	normal			*de novo*
9	p.Met504Val	normal	normal			*de novo*
10	p.Ala72Ser	normal	normal			*de novo*
11	p.Glu139Asp	normal	normal			*de novo*
12	p.Gly503Arg	normal	normal			*de novo*
13	p.Asp61Gly	normal	normal			*de novo*
14	p.Phe285Ser	normal	normal			*de novo*
15	p.Met504Val	normal	normal			*de novo*
16	p.Met504Val	normal	normal			*de novo*
17	p.Met504Val	normal	normal			*de novo*
18	p.Asn308Asp	normal	normal			*de novo*
19	p.Tyr63Cys	affected	normal	normal		Maternal
20	p.Glu69Gln	affected	normal	affected	normal	Maternal
21	p.Tyr279Cys	affected	not studied			Maternal
22	p.Tyr62Asp	affected	not studied			Maternal
23	p.Gln79Arg	affected	normal			Maternal
24	p.Tyr279Cys	affected	not studied			Maternal
25	p.Gly60Ala	not studied	affected			Maternal
26	p.Asn308Asp	normal	affected	normal		Paternal
27	p.Thr468Met	normal	affected	normal		Paternal
28	p.Asn308Asp	not studied	not studied	affected		not *de novo*
29	p.Asn308Ser	normal	not studied			Not determined
30	p.Phe285Ser	normal	not studied			Not determined
31	p.Met504Val	normal	not studied			Not determined
32	p.Asn58Asp	normal	not studied			Not determined
33	p.Asn308Asp	normal	not studied			Not determined
34	p.Met504Val	not studied	normal			Not determined
35	p.Ala72Ser	normal	not studied			Not determined
